# Chondroblastoma of the Knee Treated with Resection and Osteochondral Allograft Reconstruction

**DOI:** 10.1155/2014/543959

**Published:** 2014-12-08

**Authors:** Judd Fitzgerald, Cory Broehm, David Chafey, Gehron Treme

**Affiliations:** ^1^Orthopaedics & Rehabilitation, 1 University of New Mexico, MSC10 5600, Albuquerque, NM 87131-0001, USA; ^2^Department of Pathology, 1 University of New Mexico, MSC08 4640, BMSB, Room 335, Albuquerque, NM 87131, USA

## Abstract

*Case*. This case report describes the operative management of 16-year-old male with a symptomatic chondroblastoma of the distal femur with breach of the chondral surface. Following appropriate imaging and core needle biopsy, the diagnosis was confirmed histologically. The patient then underwent intralesional curettage and osteochondral allograft reconstruction of the defect. At one-year follow-up the patient was pain-free and has obtained excellent range of motion. There is radiographic evidence of allograft incorporation and no evidence of local recurrence. *Conclusion*. Osteochondral allograft reconstruction is an effective option following marginal resection and curettage of chondroblastoma involving the chondral surface of the distal femur.

## 1. Introduction

Chondroblastoma was first described by Codman in 1928 as “epiphyseal chondromatous giant cell tumors of the proximal humerus” [1]. This was later recognized by Jaffe and Lichtenstein in 1942 as a distinct entity [2]. Chondroblastomas most frequently affect the epiphyses of long bones, most commonly about the knee and proximal humerus [3–6]. Subchondral thinning has been reported in up to 62% of cases and chondral breach and collapse in up to 33% of cases [7]. These lesions are benign but may be histologically aggressive with rare lung metastases and a high reported recurrence rate of 8 to 38% [4, 5, 8–12]. Most recurrences occur within the first 3 years but have been reported up to 7 years after curettage [4, 5, 10]. Chondroblastomas typically present in the second decade of life with 95% of patients presenting between the ages of 5 and 25 [13] and have a 2 : 1 male-to-female predominance [13]. Common presenting symptoms include pain with activity and rest as well as local tenderness, effusion, and loss of range of motion of the affected joint [6].

## 2. Case Presentation

**Figure 1:**
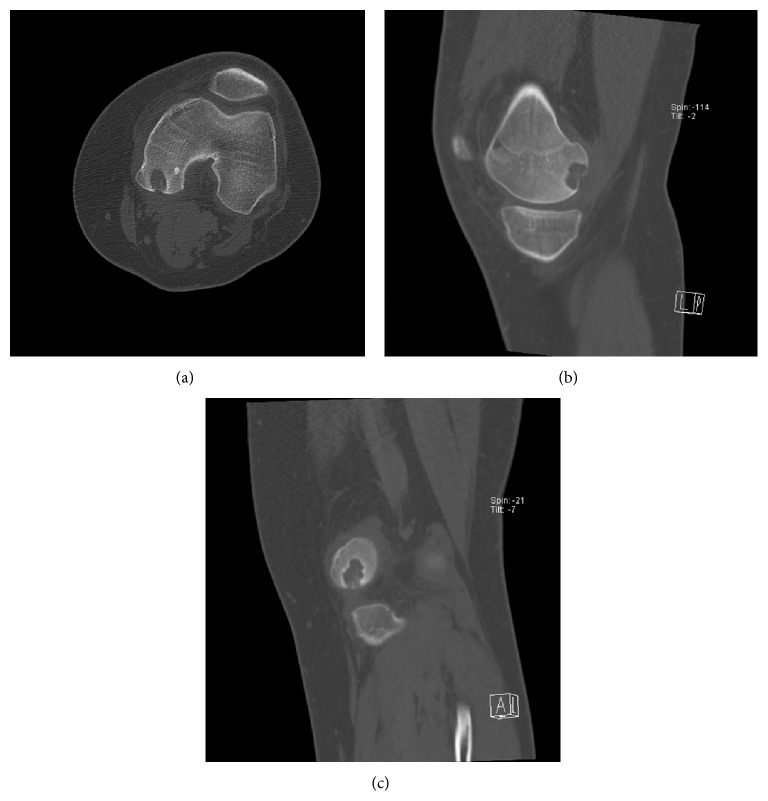
Axial (a), sagittal (b), and coronal (c) images from preoperative CT scan demonstrating a lytic lesion in the medial femoral condyle with lobulated well-defined margins and depression of the subchondral bone at the lesion border.

**Figure 2:**
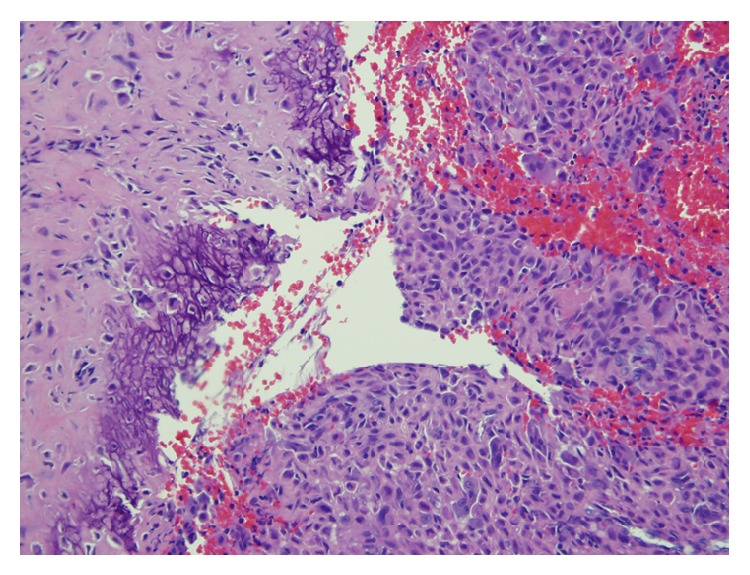
Mononuclear neoplastic cells with intermixed multinucleated giant cells (right), tumor cells surrounded by dark blue “chicken wire” calcifications (left center), and fibrochondroid islands (left) (hematoxylin and eosin stain, 20x magnification).

**Figure 3:**
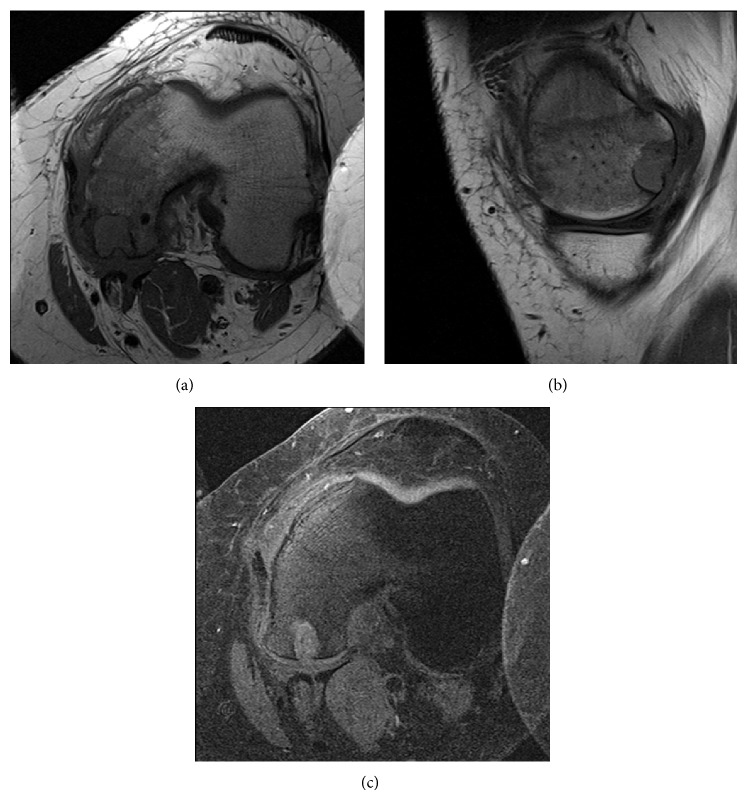
T1 TSE axial (a) and sagittal (b) images and T1 TSE w/FS axial (c) image showing a lytic lesion with sclerotic borders involving the posterior aspect of the medial femoral condyle and abutting the posterior articular surface with 3 mm collapse of the subchondral bone and breach of the chondral surface.

**Figure 4:**
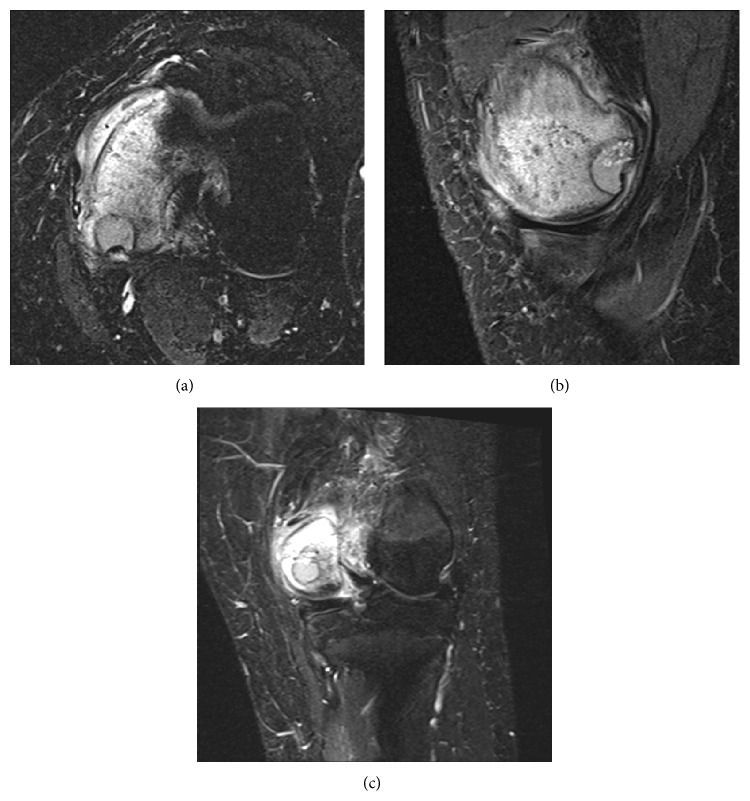
STIR axial (a), sagittal (b), and coronal (c) images demonstrating associated medial femoral condyle marrow edema and adjacent soft tissue edema and joint effusion.

In August of 2012 a 16-year-old male presented complaining of rest and activity pain worsening for the past year. He denied any history of trauma. The patient reported is worsening left knee pain and decreased range of motion were limiting his daily activities. Radiographs from an outside facility (not available for publication) showed a lobulated well-defined epiphyseal lesion with sclerotic margins and without stippled calcifications. Computerized tomography (CT) further defined a low-attenuation lesion in the medial femoral condyle measuring 13 mm by 16 mm with well-defined sclerotic margins and depression of the subchondral bone at the lesion border (Figure 1). Differential diagnosis at this time included chondroblastoma, giant cell tumor, clear cell chondrosarcoma, and osteomyelitis. Given the patient's delayed presentation and our increased concern for metastatic disease we elected to proceed with a CT chest, in place of a routine screening chest radiograph. This CT showed no evidence of metastatic disease. Core needle biopsy (Figure 2) showed mononuclear neoplastic cells with intermixed multinucleated giant cells, tumor cells surrounded by dark blue “chicken wire” calcifications, and fibrochondroid islands all consistent with a chondroblastoma. At this point an MRI of the left knee was obtained to evaluate the extent of articular cartilage involvement and to determine reconstruction options after recommended intralesional curettage. MRI demonstrated a lytic lesion with sclerotic borders involving the posterior aspect of the medial femoral condyle and abutting the posterior articular surface with a 3 mm collapse of the subchondral bone and breach of the chondral surface (Figure 3). Also the significant enhancing of marrow edema in the medial femoral condyle and adjacent soft tissue edema with associated joint effusion is well demonstrated on the T2 STIR images (Figure 4). The imaging and pathology were reviewed at multidisciplinary tumor board and the surgical approach and reconstructive options were discussed at the weekly orthopaedic departmental preoperative planning meeting. It was determined that no medical therapy was indicated as there is no role for chemotherapy in the management of chondroblastoma and radiation therapy is not recommended given an increase in risk of malignant transformation [10, 14]. Surgical plans were then made for curettage and osteochondral allograft reconstruction using a direct approach.

**Figure 5:**
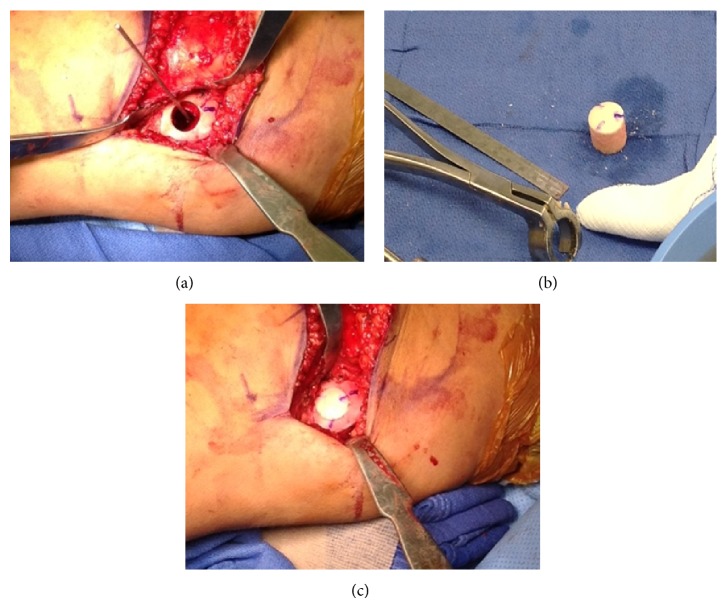
Intraoperative photographs showing marginal excision and preparation of medial femoral condyle (a), osteochondral allograft preparation (b), and implantation (c).

The patient underwent resection and allograft reconstruction in January of 2013. The posterior aspect of the medial femoral condyle was approached through a posterior medial approach to the knee using an extensile skin incision dissecting down through the interval between the medial head of the gastrocnemius and the semimembranosus tendon as described by Burks and Schaffer [15]. The lesion was identified and a high-speed burr and curette were used to achieve intralesional curettage and the tissue was sent to pathology. Then using a K-wire in the center of the lesion a 20 mm trephine was used to the depth of 25 mm in preparation for the allograft. Hydrogen peroxide irrigation and a high-speed burr were used as adjuvant therapy. A 20 mm trephine was then used to the depth of 25 mm in the articular surface of the fresh frozen distal femur allograft (obtained from the Musculoskeletal Transplant Foundation) to prepare the osteochondral plug. The graft was then inserted in a press fit fashion using clock face markings for orientation (Figure 5). Stability of the allograft was then assessed and confirmed. The wound was irrigated and closed in standard fashion.

**Figure 6:**
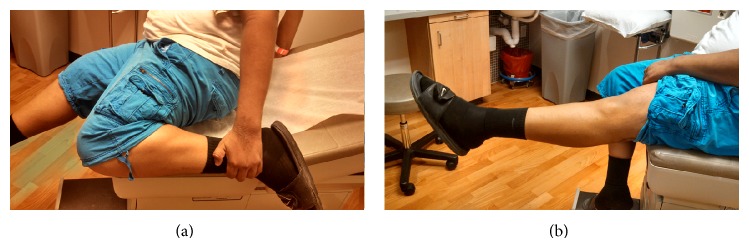
Five-month postoperative photos demonstrating full flexion (a) and extension (b).

**Figure 7:**
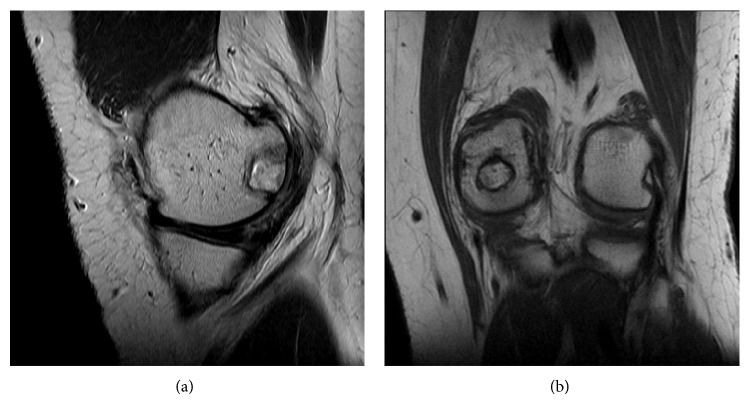
One-year postoperative PD TSE sagittal (a) and T1 TSE coronal (b) images showing osteochondral allograft incorporation and congruency of chondral surface. No evidence of local recurrence.

**Figure 8:**
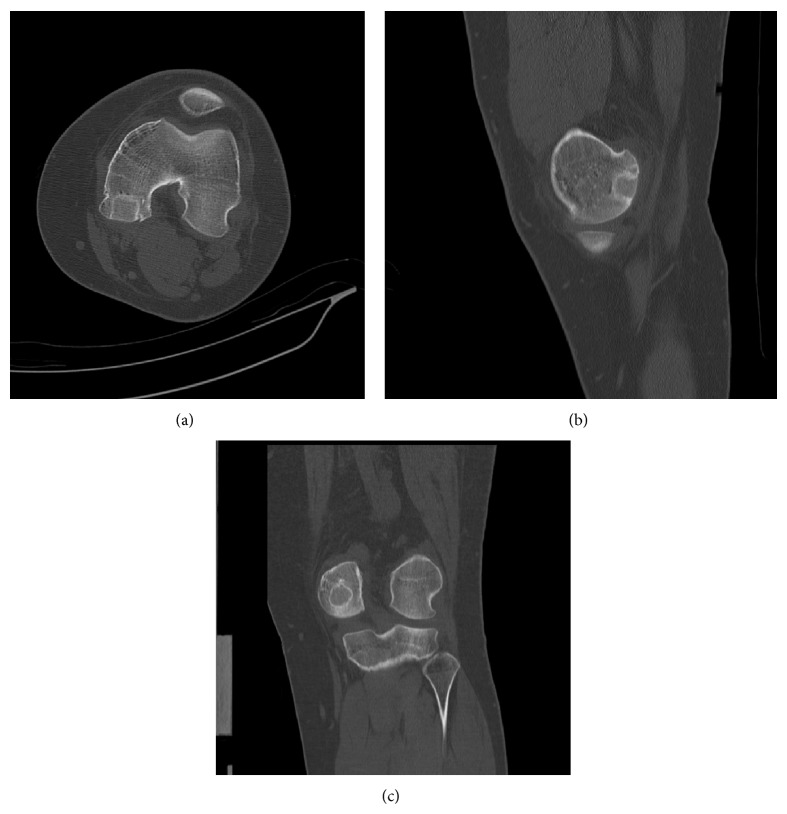
One-year postoperative axial (a), sagittal (b), and coronal (c) CT scan demonstrating osteochondral allograft incorporation and congruency of chondral surface. No evidence of local recurrence.

The patient tolerated the procedure well without complication and was instructed to remain touch down weight bearing in a hinged knee brace locked in full extension for two weeks. He was seen two weeks postoperatively with minimal pain and swelling noted on exam. At this point the Bledsoe brace was unlocked and he was allowed to weight bear as tolerated. He then completed a full course of physical therapy and was seen in follow-up at the three- and five-month marks. At the five-month postoperative visit he denied any pain and reported ability to run and jump without pain or limitation. He demonstrated full active flexion and extension compared to the contralateral extremity (Figure 6). At his one-year anniversary appointment he again denied pain and demonstrated full painless range of motion, similar to the contralateral knee. He had no effusion and no tenderness to palpation over the medial femoral condyle. MRI and CT imaging of the left knee were obtained which demonstrated incorporation of the osteochondral allograft (Figures 7 and 8) and resolution of the previously associated medial condyle marrow edema. MRI and CT scan of the knee and CT chest also revealed no evidence of local recurrence or distant metastasis, respectively.

## 3. Discussion

Chondroblastoma is a rare benign primary bone tumor commonly presenting in the knee of young patients [3, 5]. Given the location and possible involvement of the articular surface of the knee and the physical demands of this demographic this benign bone tumor and its conventional surgical treatment can be a significant source of morbidity. Historically, given the rare reported incidents of metastases and their potential to be locally aggressive, treatment has consisted of intralesional curettage and bone grafting versus wide resection and reconstruction. Lesions that significantly involve articular cartilage present a surgical challenge when trying to maintain the integrity and function of the joint. Though effective, these reconstruction options have high reported complication rates largely related to loss of joint integrity and stability, weakness, stiffness, joint arthrosis, and recurrence [6, 10, 16, 17].

We report a case of chondroblastoma involving the posterior surface of the medial femoral condyle in an otherwise healthy and active 18-year-old male. After obtaining appropriate imaging and a tissue diagnosis via core needle biopsy, marginal excision and osteochondral allograft reconstruction was performed in an attempt to prevent recurrence and maintain joint integrity. At the one-year mark there was no evidence of disease recurrence and the patient has achieved full painless, active range of motion. He has returned to school and sport and is very satisfied with the outcome of his treatment. At one year the osteochondral allograft has incorporated and remained stable and flush with the remaining articular surface, which should limit the progression of the degenerative process. At this point we will continue to follow up the patient for evidence of local recurrence, metastasis, and degenerative change with serial radiographs. We would expect our patient's risk of local recurrence and metastatic disease to be similar to rates reported in the literature as we treated the lesion with standard intralesional curettage and local adjuvant therapy. Our focus, as it relates to this case report, will be long-term follow-up of osteochondral allograft incorporation and progression of degenerative disease.

Given our experience with this case we feel that intralesional curettage with osteochondral allograft reconstruction is a viable surgical option for chondroblastomas involving the articular surface of the knee. This reconstruction technique allows for painless weight bearing and full range of motion.
